# Albumin knockout mice exhibit reduced plasma free fatty acid concentration and enhanced insulin sensitivity

**DOI:** 10.14814/phy2.15161

**Published:** 2022-03-03

**Authors:** Afsoun Abdollahi, Brianna N. Dowden, Kimberly K. Buhman, Alyssa S. Zembroski, Gregory C. Henderson

**Affiliations:** ^1^ Department of Nutrition Science Purdue University West Lafayette Indiana USA

**Keywords:** albumin deficiency, analbuminemia, nonesterified fatty acid, steatosis

## Abstract

Circulating albumin is expected to play a significant role in the trafficking of plasma free fatty acids (FFA) between tissues, such as FFA transfer from adipose tissue to the liver. However, it was not yet known how disrupting FFA binding to albumin in circulation would alter lipid metabolism and any resulting impacts upon control of glycemia. To improve understanding of metabolic control, we aimed to determine whether lack of serum albumin would decrease plasma FFA, hepatic lipid storage, whole body substrate oxidation, and glucose metabolism. Male and female homozygous albumin knockout mice and C57BL/6J wild type controls, each on a standard diet containing a moderate fat content, were studied at 6–8 weeks of age. Indirect calorimetry, glucose tolerance testing, insulin tolerance testing, exercise performance, plasma proteome, and tissue analyses were performed. In both sexes of albumin knockout mice compared to the wild type mice, significant reductions (*p* < 0.05) were observed for plasma FFA concentration, hepatic triacylglycerol and diacylglycerol content, blood glucose during the glucose tolerance test, and blood glucose during the insulin tolerance test. Albumin deficiency did not reduce whole body fat oxidation over a 24‐h period and did not alter exercise performance in an incremental treadmill test. The system‐level phenotypic changes in lipid and glucose metabolism were accompanied by reduced hepatic perilipin‐2 expression (*p* < 0.05), as well as increased expression of adiponectin (*p* < 0.05) and glucose transporter‐4 (*p* < 0.05) in adipose tissue. The results indicate an important role of albumin and plasma FFA concentration in lipid metabolism and glucoregulation.

## Graphical abstract

Plasma free fatty acids (FFA) bind to albumin in the bloodstream to facilitate their transport from adipose tissue to other sites in the body. Albumin knockout mice exhibited reduced plasma FFA concentration, reduced hepatic lipids (triacylglycerol and diacylglycerol), and improved insulin sensitivity. The results indicate that disrupting the trafficking of FFA between tissues, through disruption of albumin expression, may lead to altered lipid metabolism and maintenance of lower blood glucose concentration.

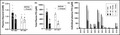

## 1 INTRODUCTION

Excess energy in the body is primarily stored as triacylglycerol (TAG) in adipose tissue. In healthy individuals, rapid mobilization of free fatty acids (FFA) into circulation, through TAG lipolysis and FFA export from adipose tissue, facilitates the use of FFA as an alternative fuel. Use of FFA is believed to spare blood glucose, and the capacity to achieve high plasma FFA concentrations likely evolved for defense of glycemia when stresses such as exercise or fasting are imposed (Brooks et al., 2005; Green et al., 2011; Henderson et al., 2007; Hogild et al., 2019; Johnson et al., 2006). While maintaining substantial levels of plasma FFA is believed to play a role in physiological performance, there is also strong evidence that chronic reduction of plasma FFA concentration can actually improve health. Plasma FFA concentration is a modifiable risk factor for a wide variety of metabolic health problems, with lower levels corresponding to superior health status (Henderson, 2021). For example, chronic physical inactivity (Paolisso et al., 1997; Smart et al., 2018), obesity (Fabbrini et al., 2009; Gastaldelli et al., 2009; Hagman et al., 2019; Jansson et al., 1998; Jensen et al., 1989; Pardina et al., 2009), sleep apnea (Barcelo et al., 2011; Jun et al., 2011), and insufficient sleep quantity (Broussard et al., 2015; Rao et al., 2015) are each associated with elevated plasma FFA concentration and poor insulin sensitivity. Even in the absence of overt pathology, still plasma FFA reduction may be useful for achieving a relative health improvement. For example, experimental reductions of plasma FFA concentration through administration of nicotinic acid or acipimox reduces plasma FFA concentration (Ali et al., 2015; Gorski et al., 1988; Hu et al., 2012; Paolisso et al., 1997) and consequently improves insulin sensitivity (Bajaj et al., 2005; Blachere et al., 2001; Hadigan et al., 2003; Paolisso et al., 1997; Santomauro et al., 1999), accompanied by reduced hepatic lipid storage (Ali et al., 2015; Gorski et al., 1988; Hu et al., 2012). However, metabolic response to these compounds are not sustained in the long term (Dunbar & Goel, 2016; Goel & Dunbar, 2016), and new avenues for modulating plasma FFA concentration are needed. Mechanisms for the relationship between plasma FFA concentration and insulin sensitivity could be related to the level of ectopic lipid deposition. For example, TAG accumulation in liver and muscle, which is often related to the plasma FFA level, is typically associated with elevation of lipotoxic intermediates such as diacylglycerol (DAG; Camporez et al., 2015; Hall et al., 2014; Leavens et al., 2009; Puri et al., 2007; Wendel et al., 2010). DAG (and other lipotoxic intermediates such as ceramide) are inversely associated with insulin sensitivity, and reducing lipotoxic intermediates is expected to improve insulin sensitivity (Camporez et al., 2015; Perry et al., 2014; Samuel & Shulman, 2018).

Currently, there are no effective clinical treatments to reduce plasma FFA concentration or to reduce FFA uptake into tissues such as liver or muscle. Each of the metabolic steps involved in the transfer of fatty acids from the adipose tissue TAG pool to other metabolic tissues could be considered as a potential target for disrupting the redistribution of lipid from adipose tissue to ectopic lipid deposition sites. For example, hypothetically investigators could target lipolysis, FFA export through transporters such as CD36, binding of FFA to serum albumin, or uptake of FFA through FFA transporters in tissues such as liver. Previously adipose tissue lipolysis has been targeted in drug development; however, these efforts have not yet led to an approved drug (Dunbar & Goel, 2016; Goel & Dunbar, 2016). In research on gene knockout mice, while whole body adipose triglyceride lipase (ATGL) knockout mice exhibited reduced plasma FFA concentration, elevated liver and muscle steatosis levels were observed, as well as cardiac dysfunction (Haemmerle et al., 2006). While sequestering TAG within adipose tissue might be beneficial, the sequestration of lipid at ectopic lipid deposition sites in this whole body ATGL deficiency model led to pathology. However, the adipose‐specific ATGL knockout mouse showed a favorable phenotype of reduced plasma FFA, decreased hepatic lipid, and improved insulin sensitivity (Schoiswohl et al., 2015; Wu et al., 2012). Thus, plasma FFA reduction, especially when it can lead to reduced ectopic lipid deposition, still appears to be a useful approach for metabolic health improvement.

To understand the role of plasma FFA trafficking in metabolic health, it would be useful to consider other steps in the FFA mobilization process as well, such as those which are downstream of lipolysis, including FFA export from adipose tissue and binding with albumin. FFA are hydrophobic and are believed to rely upon albumin for their dissolution in the bloodstream (Fang et al., 2006; Simard et al., 2005). Thus, the efficiency of FFA export from adipose tissue is likely linked to the capacity for binding to albumin. Our goal was to test the role of albumin in determining plasma FFA concentration, effects on lipid metabolism, and impacts upon glucose metabolism and substrate oxidation. We sought to understand the potential metabolic benefits of reducing plasma FFA by targeting albumin. We designed the present study to comprehensively determine the phenotype of albumin knockout mice at the level of gene expression in metabolic tissues, in vivo physiology, and plasma proteome remodeling. These results provide a characterization of this mouse model which sets the stage for future translational research with this model to learn about interactions between albumin, plasma FFA, and metabolic health in various circumstances.

## 2 METHODS

### 2.1 Animals

This protocol was approved by the Purdue University Animal Care and Use Committee. Male and female homozygous albumin knockout mice (Alb^−/−^) on the C57BL/6J background were purchased from the Jackson Laboratory (Bar Harbor, ME); the gene ablation was a whole body knockout model (Roopenian et al., 2015). As albumin is normally expressed only in the liver, the impact of gene ablation was upon the liver. C57BL/6J control mice (wild type, WT) were also purchased from Jackson Laboratory. Six male Alb^−/−^ mice, six female Alb^−/−^ mice, six male WT mice, and six female WT mice were housed in an animal facility at Purdue University and maintained on a 12‐h light/dark photoperiod. Mice were allowed ad libitum access to food and water except for brief withdrawal of food before specific procedures as described below. All mice consumed the Labdiet 5K52 diet (Purina Mills) after weaning at 3 weeks of age for their lifetimes and were acclimated to the facility for at least 1 week prior to any experiments. Food intake was monitored at approximately 8 weeks of age. As described below, metabolic assessments were performed in the final 2 weeks of life and mice were euthanized at 8 weeks of age.

### 2.2 Oral glucose tolerance test

Oral glucose tolerance testing (OGTT) was performed at approximately 7 weeks of age, following a 4–6 h fast. Glucose was administered orally (2 g/kg bodyweight) as a 20% wt/vol solution. Blood glucose was measured in a drop of blood from the tail using a point of care device (Prodigy Diabetes Care) at times 0, 10, 20, 30, 60, 90, and 120 min. Area under the curve (AUC) was calculated as a Riemann Sum (i.e., by the trapezoidal rule), and incremental AUC was calculated by factoring out the baseline blood glucose level from the AUC calculation.

### 2.3 Insulin tolerance test

Insulin tolerance testing (ITT) was performed on the same mice (*n* = 6 mice per group) at approximately 8 weeks of age, following a 4–6 h fast. Insulin (0.75 U per kg bodyweight) was administered by intraperitoneal injection. Blood glucose was measured in a drop of blood from the tail using a point of care device (Prodigy Diabetes Care) at times 0, 10, 20, 30, 60, 90, and 120 min. AUC and incremental AUC were calculated as described above.

### 2.4 Indirect calorimetry

At approximately 7 weeks of age, indirect calorimetry assessment was performed on each mouse using an Oxymax System (Columbus Instruments). Mice were allowed free access to food and water, while oxygen consumption (VO_2_) and carbon dioxide production (VCO_2_) were measured over a 24‐h period, after an initial 24‐h acclimatization period in the metabolic cage. The respiratory quotient (RQ) was calculated as VCO_2_/VO_2_, and standard equations were used to calculate energy expenditure as well as percent of energy expenditure from fat and carbohydrate. For the purposes of the calculations, the contribution of protein to fuel oxidation was assumed to be negligible and thus assigned a zero value.

### 2.5 Exercise performance test

To determine if albumin deficiency led to an altered ability to exercise, time‐to‐exhaustion in an incremental exercise test was assessed at approximately 8 weeks of age. A rodent treadmill (Columbus Instruments) was set to a 25° incline, and the shock grid set at a low intensity (on a scale of 0–10, set at 1). Exhaustion was defined as a mouse remaining on the shock grid for 5 s. The treadmill speed was started at 6 m/min for a duration of 5 min, and then increased by 3 m/min at each subsequent 2 min interval. When a mouse reached exhaustion, the time was noted and the mouse was removed from the test.

### 2.6 Tissue collection

Food was withdrawn for 4–6 h before euthanasia. Mice were euthanized by CO_2_ inhalation followed by immediate collection of blood via cardiac puncture. Blood was collected in ethylenediaminetetraacetic acid (EDTA)‐coated tubes, centrifuged at 3260× g for 10 min at 4°C, followed by isolation, and storage of plasma at −80°C until analysis. Liver, skeletal muscle, and the gonadal fat pad (epididymal adipose tissue, eWAT) were quickly collected following blood draw, immediately frozen in liquid nitrogen then stored at −80°C until analysis.

### 2.7 Lipid assays

For FFA analysis, lipids were extracted from plasma with heptane, isopropanol, and 0.033N sulfuric acid. Heptadecanoic acid (17:0) was used as the internal standard, and heptane extracts were analyzed by liquid chromatography/mass spectrometry (LC‐MS) using a model 1260 LC and model 6160 single‐quadrupole MS (Agilent Technologies); reversed phase separation and selective ion monitoring were employed to quantitate the internal standard, myristic acid (14:0), myristoleic acid (14:1), palmitic acid (16:0), palmitoleic acid (16:1), stearic acid (18:0), oleic acid (18:1), linoleic acid (18:2), α‐linolenic acid (18:3), arachidonic acid (20:4), eicosapentaenoic acid (20:5), and docosahexaenoic acid (22:6) following our established method (Davitt et al., 1985; Henderson, 2013; Liou et al., 2013). Plasma glycerol and TAG (Millipore‐Sigma), as well as plasma total cholesterol (TC) and plasma high‐density lipoprotein cholesterol (HDL‐C; Pointe Scientific) were measured using commercially available kits with a Biotek Epoch 2 plate reader (Agilent Technologies). For measurement of TAG concentration in liver and muscle, a small piece of each tissue was processed in a bead homogenizer and extracted with heptane/isopropanol/water. The heptane phase was dried and then analyzed by a colorimetric TAG assay kit (Millipore‐Sigma) and a Biotek Epoch 2 plate reader (Agilent Technologies). As liver tissue showed significant group differences for TAG content, the lipotoxic intermediates DAG, and ceramide were next measured in new extracts from this tissue, and cholesterol ester (CE) was also measured as it is an additional storage form of lipid in the liver; for these analyses, liver tissue was processed in a bead homogenizer and extracted with chloroform/methanol/water. For DAG analysis, diheptadecanoyl‐DAG was used as the internal standard, and the chloroform phase was dried and then analyzed with reversed phase LC separation and detection by a model 6410 tandem mass spectrometer (Agilent Technologies) using multiple reaction monitoring (MRM) based upon the LipidMaps database. For ceramide and CE, chloroform extracts were analyzed, with addition of [^7^H_2_] pentadecanoyl‐ceramide internal standard for ceramide and addition of [^7^H_2_] oleoylcholesterol internal standard for CE analysis; analyses of ceramide and CE were by direct infusion into a 6410 model tandem mass spectrometer (Agilent Technologies) and MRM based upon the LipidMaps database.

### 2.8 mRNA quantitation

Approximately 100 mg of adipose tissue (gonadal fat pad), 10 mg of liver, and 30 mg of skeletal muscle (triceps) were each processed with a bead homogenizer in Trizol Reagent. RNA was subsequently isolated with RNA isolation kits (Qiagen). cDNA was synthesized from DNase‐treated RNA using the AffinityScript qPCR cDNA Synthesis Kit (Agilent Technologies). SYBR green qPCR was performed using a QuantStudio 7 Real‐Time PCR System (Applied Biosystems) and PowerUp SYBR Green Master Mix (Applied Biosystems). Primers used for determination of relative mRNA levels was produced by Integrated DNA Technologies and are listed in Table 1. All samples were analyzed in triplicate with 18S ribosomal RNA as the control and results are expressed as arbitrary units with calculations by the comparative Ct method.

**TABLE 1:** Primers for qPCR

Gene	Primer sequences
Adiponectin	F 5′‐CGTTACTACAACTGAAGAGCTA‐3′
	R 5′‐TCCTGTCATTCCAACATCTC‐3′
LPL	F 5′‐TGGAATCCAGAAACCAGTAGGGCA‐3′
	R 5′‐TCTACAACTCAGGCAGAGCCCTTT‐3′
Glut4	F 5′‐GAGCTGGTGTGGTCAATA‐3′
	R 5′‐GGAGGACGGCAAATAGAA‐3′
DGAT1	F 5′‐ACCGCGAGTTCTACAGAGATTGGT‐3′
	R 5′‐ACAGCTGCATTGCCATAGTTCCCT‐3′
DGAT2	F 5′‐TGGGTCCAGAAGAAGTTCCAGAAGTA‐3′
	R 5′‐ACCTCAGTCTCTGGAAGGCCAAAT‐3′
MTP	F 5′‐AGTGCAGTTCTCACAGTACCCGTT‐3′
	R 5′‐AGCATATCGTTCTGGTGGAAGGGA‐3′
Plin2	F 5′‐CTTGTGTCCTCCGCTTAT‐3′
	R 5′‐TGCTCCTTTGGTCTTATCC‐3′
CD36	F 5′‐GGCCAAGCTATTGCGACATG‐3′
	R 5′‐CCGAACACAGCGTAGATAGAC‐3′
FSP27	F 5′‐TCGGAAGGTTCGCAAAGGCATC‐3′
	R 5′‐CTCCACGATTGTGCCATCTTCC‐3′
18S	F 5′‐TTAGAGTGTTCAAAGCAGGCCCGA‐3′
	R 5′‐TCTTGGCAAATGCTTTCGCTCTGG‐3′

### 2.9 Western blotting and ELISA

Approximately 40 mg of liver was homogenized using a bead homogenizer in 20 volumes Cell Lysis Buffer (Cell Signaling Technology) containing Complete Mini Protease Inhibitor (Roche) and Halt Phosphatase Inhibitor Cocktail (Thermo Scientific). The homogenate was centrifuged at 10,000 *g* for 15 min at 4°C and the protein content of the supernatant was determined by the Bradford method (Bradford, 1976) using a commercially available reagent (BioRad). The supernatant was then diluted to a total protein concentration of 2 µg/µl with a 19:1 vol/vol mixture of Laemmli Sample Buffer (BioRad) and beta‐mercapto ethanol (Millipore‐Sigma) then heated at 95°C for 5 min to denature proteins. The denatured protein mixture was loaded onto a 4%–15% polyacrylamide gel (BioRad). Proteins were separated by electrophoresis, transferred onto a nitrocellulose membrane (BioRad), then blocked for 1 h at room temperature with Tris‐buffered saline (TBS; BioRad) containing 5% nonfat dried milk. Primary antibodies were diluted in TBST (TBS containing 0.1% tween 20; BioRad) with 1% nonfat dried milk. Membranes were incubated overnight at 4°C with primary antibodies against fatty acid translocase (CD36; 1:500; Fisher Scientific), fat‐specific protein 27 (FSP27; 1:1000; Novus Biologicals), microsomal triglyceride transfer protein (MTP; 1:5000; BD Biosciences), perilipin‐2 (PLIN2; 1:4000; kindly provided by Dr. Perry Bickel of the University of Texas Southwestern Medical Center), fatty acid synthase (FASN; 1:1000; Cell Signaling Technology), ATP Citrate Lyase (ACLY; 1:1000; Protein Tech), total ACC (1:8000; Abcam), phospho‐ACC^Ser79^ (1:1000; Cell Signaling Technology), and β‐actin (1:1000; Cell Signaling Technology). Membranes were then washed with TBST, then incubated at room temperature for 1 h with IR Dye 680 (1:15,000; LI‐COR Biosciences) or IR Dye 800 (1:15,000; LI‐COR Biosciences) secondary antibodies dissolved in TBST containing 1% nonfat dried milk and 0.02% SDS (BioRad). Membranes were then washed with TBST and bands were visualized and quantified using a LI‐COR Odyssey Infrared Imaging System (LI‐COR Biosciences) with values normalized to β‐actin bands as the loading control. Insulin was measured in plasma by enzyme‐linked immunosorbent assay (Merdodia) in the Indiana University Translation Core.

### 2.10 Proteomics analysis

Protein concentration in plasma was determined by the Bradford method (Bradford, 1976) using a commercially available reagent (BioRad). An equal volume of each plasma sample (1 µl) was prepared for global proteomic analysis; proteins were reduced using 10 mM dithiothreitol and then alkylated with 20 mM iodoacetamide prior to digestion with 4 mg Trypsin/Lys‐C Mix (Promega). Samples were cleaned using MicroSpin TM C18 reversed phase spin columns (The Nest Group). Cleaned peptides were dried using a vacuum centrifuge, resuspended in 3% acetonitrile/0.1% formic acid and loaded into the mass spectrometer for LC‐MS/MS analysis using 2 h LC gradients. Analysis was performed using a Dionex UltiMate 3000 RSLC nano System coupled on‐line to Orbitrap Fusion Lumos Mass Spectrometer (Thermo Fisher Scientific) at the Purdue Proteomics Facility. Reverse phase peptide separation was accomplished using a trap column (300 mm ID × 5 mm) packed with 5 mm 100 Å PepMap C18 medium coupled to a 50‐cm long × 75 µm inner diameter analytical column packed with 2 µm 100 Å PepMap C18 silica (Thermo Fisher Scientific). The data were processed using MaxQuant software against the UniProtKB Mus musculus protein database. Results were filtered at 1% false discovery rate for both peptides and proteins, and results quantified using label‐free quantitation precursor ion (MS1) intensity. Proteins with greater than a single zero value in WT males or females were removed from the dataset. Proteins with a fold change of 1.5 or greater were selected, and additional filtering was performed by ANOVA as described below.

### 2.11 Statistical analysis

Data are presented as means ± SE. The results were analyzed by two‐way ANOVA, with genotype (WT vs. Alb^−/−^) and sex as the factors. Results were also analyzed by three‐way ANOVA, when appropriate, including time as a factor. For analysis of VO_2_, metabolic rate, Lox, and CHOox, the two‐way and three‐way analysis were carried out as an ANCOVA with body weight set as the covariate. ANOVA and ANCOVA were followed by Fisher's least significant difference post hoc test when appropriate. Statistical analyses were performed with JMP version 14 (SAS Institute Inc.) and *p* ≤ 0.05 was considered statistically significant.

## 3 RESULTS

**FIGURE 1:**
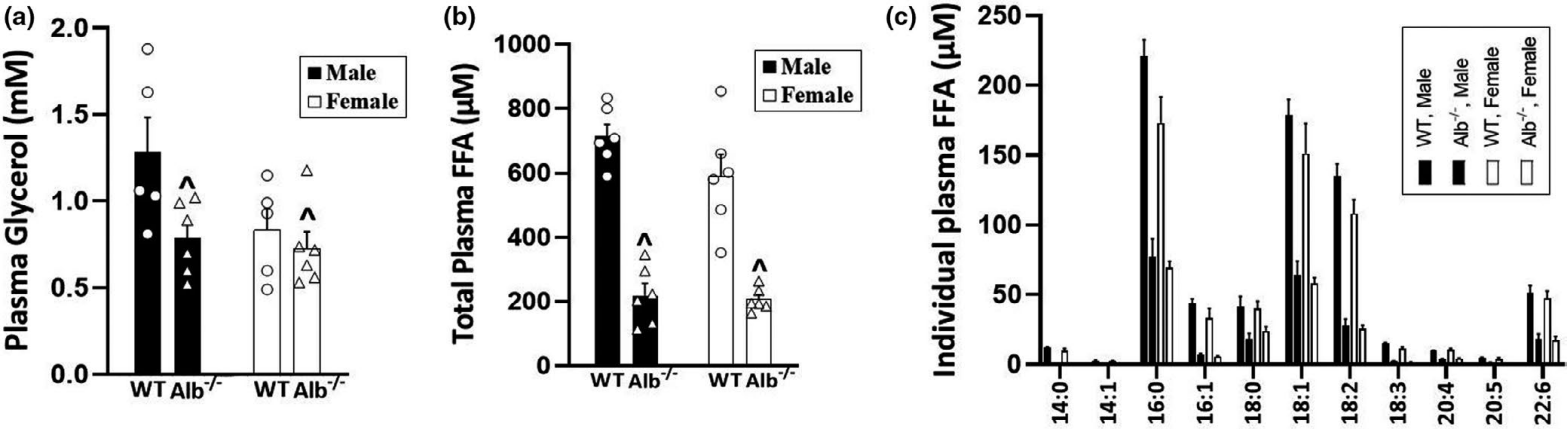
Plasma glycerol concentration (a), total plasma free fatty acid (FFA) concentration (b), and concentration of individual FFA species in plasma (c). *n* = 6 mice per group for FFA and *n* = 5–6 per group for glycerol. Analysis by ANOVA. ^Alb^−/−^ significantly different from WT (main effect of genotype, *p* < 0.05). The order of histogram bars in (c) is the same as (a) and (b) (i.e., from left to right for each FFA: WT male, Alb^−/−^ male, WT female, Alb^−/−^ female). Significant main effect of genotype was observed for each individual FFA species as well (c), indicating significantly lower FFA concentration in Alb^−/−^ than WT; due to space considerations in this panel, the symbols are not shown graphically

For total plasma FFA (Figure 1b), the ANOVA indicated a significant main effect of genotype (*p* < 0.05) with no significant main effect of sex or interaction. The main effect of genotype corresponded to lower plasma FFA in Alb^−/−^ than WT. Each individual FFA that was measured (14:0, 14:1, 16:0, 16:1, 18:0, 18:1, 18:2, 18:3, 20:4, 20:5, 22:6) was significantly lower in Alb^−/−^ than WT for both males and females (*p* < 0.05; Figure 1c). There was also a significant main effect of genotype for plasma glycerol (*p* < 0.05) with no significant main effect of sex or interaction (Figure 1a). For plasma TAG, there were no significant main effects of genotype or sex, nor any significant interaction (WT male, 1.0 ± 0.2, Alb^−/−^ male, 1.6 ± 0.4, WT female, 0.8 ± 0.02, Alb^−/−^ female, 1.1 ± 0.2 mM). Plasma TC (WT male, 105 ± 7, Alb^−/−^ male, 146 ± 18, WT female, 93 ± 4, Alb^−/−^ female, 165 ± 9 mg/dl) and HDL‐C (WT male, 41 ± 5, Alb^−/−^ male, 49 ± 6, WT female, 29 ± 3, Alb^−/−^ female, 50 ± 4 mg/dl) each exhibited a main effect of genotype (*p* < 0.05) with no significant main effect of sex or interaction. The ratio between HDL‐C and TC was not significantly different between genotypes. Food intake and exercise capacity are reported as animal characteristics in Table 2. Body weight was not different between Alb^−/−^ and WT (no significant main effect of genotype), but it was significantly higher in males than females (main effect of sex, *p* < 0.05), and there was no significant sex‐by‐genotype interaction. The eWAT fat pad was weighed after necropsy and was not different between Alb^−/−^ and WT (no significant main effect of genotype). The eWAT weight was significantly higher in males than females (main effect of sex, *p* < 0.05), and there was no significant sex‐by‐genotype interaction. Food intake was normalized to body weight and was not different between Alb^−/−^ and WT (no significant main effect of genotype). Food intake was significantly higher in females than males (main effect of sex, *p* < 0.05), and there was no significant sex‐by‐genotype interaction. Exercise capacity (time‐to‐exhaustion in the excremental exercise test) was not different between Alb^−/−^ and WT (no significant main effect of genotype). Exercise capacity was significantly greater in females than males (main effect of sex, *p* < 0.05), and there was no significant sex‐by‐genotype interaction.

**TABLE 2:** Animal characteristics

	Male		Female	
	WT	Alb^−/−^	WT	Alb^−/−^
Body weight (g)*	23.4 ± 0.6	21.4 ± 1.0	18.4 ± 0.3	18.1 ± 0.4
eWAT weight (g)*	0.27 ± 0.02	0.30 ± 0.05	0.12 ± 0.02	0.19 ± 0.03
Food intake (g/gBW/wk)*	1.4 ± 0.1	1.3 ± 0.1	1.7 ± 0.1	1.6 ± 0.1
Exercise capacity (min)*	11.0 ± 1.0	12.0 ± 0.8	15.8 ± 1.8	14.1 ± 0.7

Note

Values are means ± SE. Food intake expressed as grams of food per day, normalized to grams of body weight. Exercise capacity expressed as time‐to‐exhaustion in an incremental exercise test on a treadmill. Statistical analysis by ANOVA. Main effect of sex (male different from female), *p < 0.05.

Abbreviation: eWAT, epididymal (gonadal) white adipose tissue.

**FIGURE 2:**
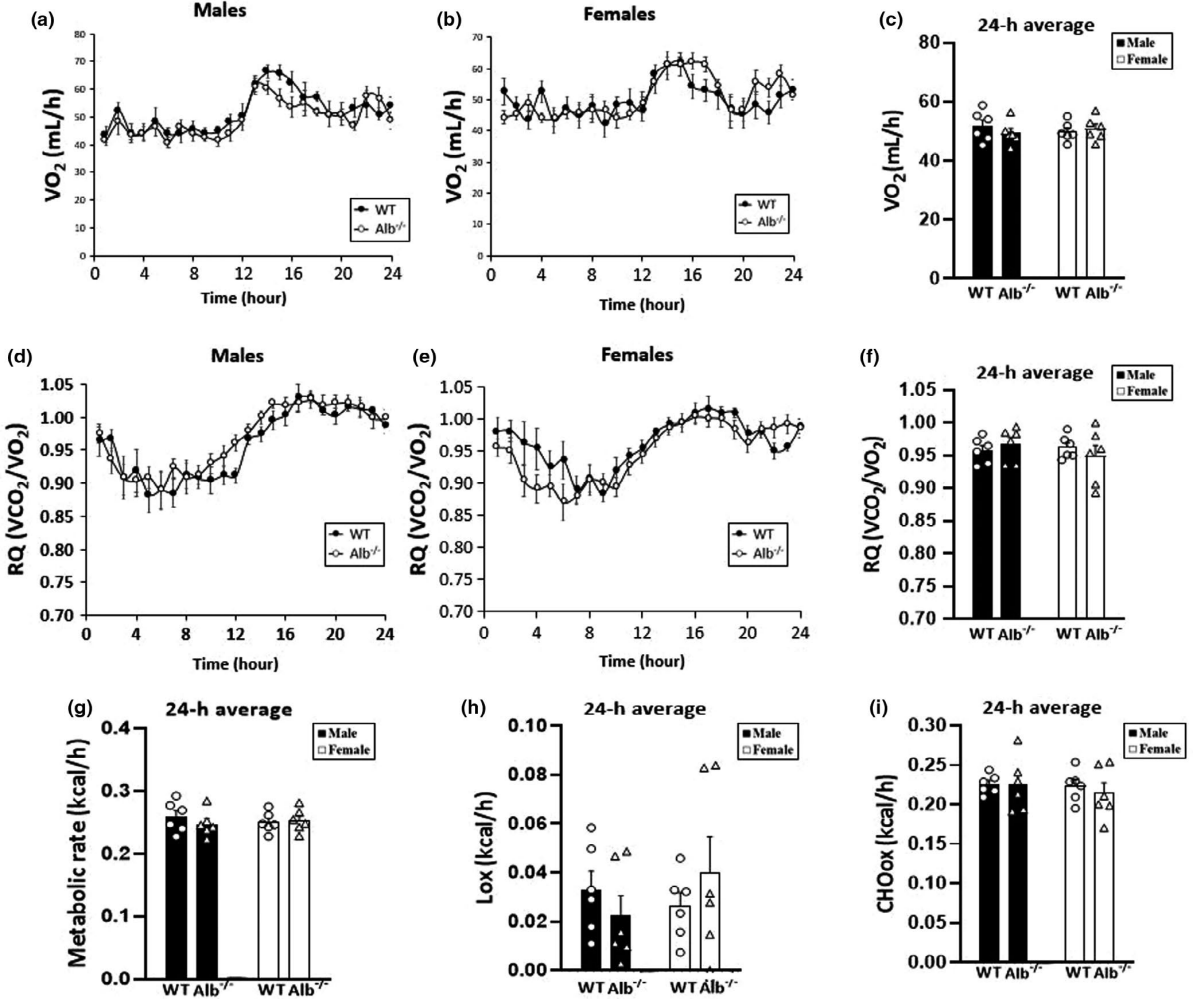
Indirect calorimetry results. (a–c) Oxygen consumption (VO_2_); (d–f) Respiratory quotient (RQ); (g) 24‐h average of metabolic rate, (h) 24‐h average of lipid oxidation (Lox), and (i) 24‐h average of carbohydrate oxidation (CHOox). *n* = 6 mice per group. Analysis of RQ by ANOVA. Analysis of VO_2_, metabolic rate, Lox, and CHOox by ANCOVA with body weight as the covariate

To determine if reduced plasma FFA carrying capacity would be associated with a reduced metabolic rate or reduced fat oxidation, we performed indirect calorimetry analyses on live mice. Indirect calorimetry results for the average values over 24 h for oxygen VO_2_ (Figure 2c), RQ (Figure 2f), metabolic rate (Figure 2g), Lox (Figure 2h), and CHOox (Figure 2i) suggested that neither metabolic rate nor fat oxidation were significantly different between Alb^−/−^ and WT mice; in these two‐way ANCOVA analyses, there were no significant main effects of sex, genotype, or interaction. The three‐way ANCOVA analysis over time for VO_2_ (Figure 2a,b), RQ (Figure 2d,e), and for metabolic rate and substrate oxidation rates (data not shown) similarly indicated no significant main effect of genotype nor any significant interaction between genotype and other factors. It appeared that both metabolic rate and Lox were reasonably similar between genotypes despite the substantial differences between groups for plasma FFA concentration. Even though plasma FFA is a fuel for substrate oxidation, decreased plasma FFA concentration in albumin knockout mice did not impair metabolic rate or Lox.

**FIGURE 3:**
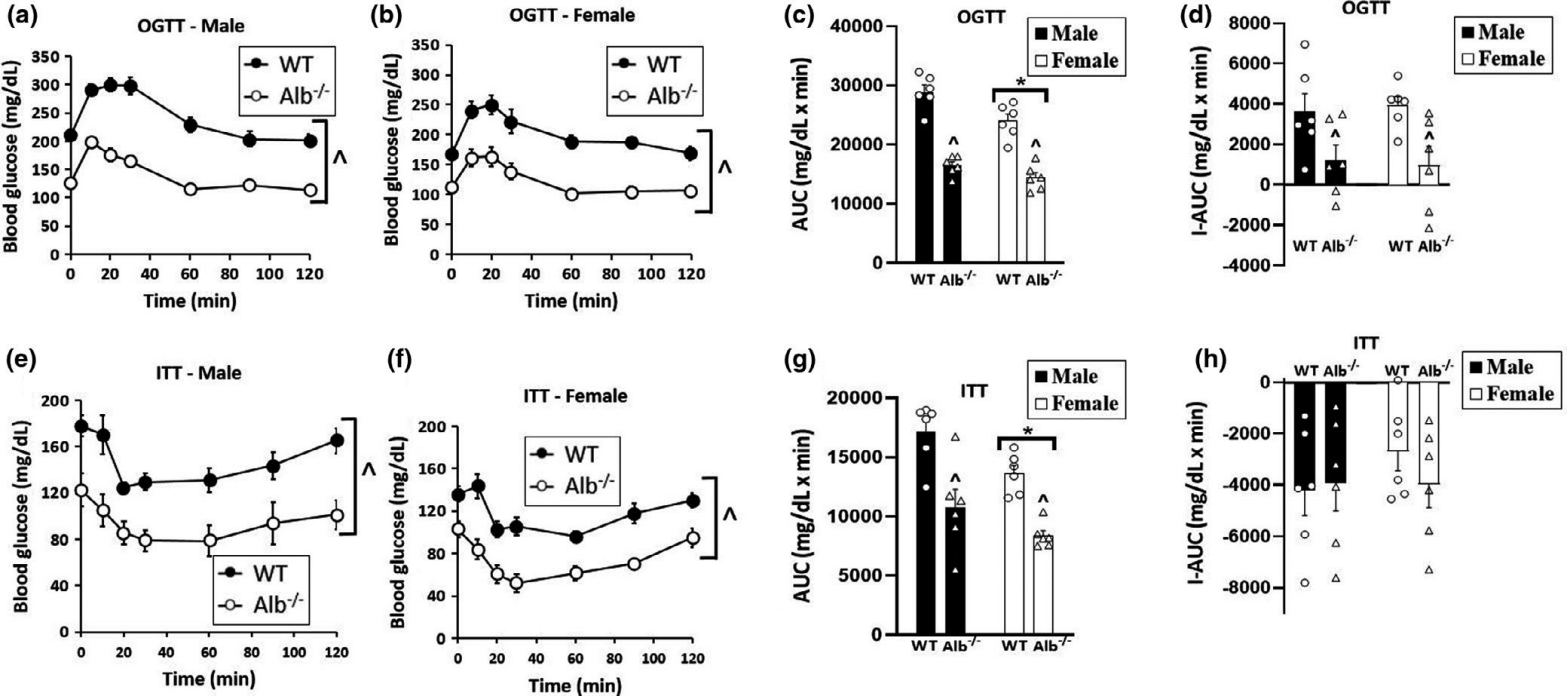
Oral glucose tolerance test (OGTT) and insulin tolerance test (ITT). Blood glucose over time for OGTT (a, b) and ITT (e, f). AUC, area under the curve for blood glucose concentration (c, g). I‐AUC, incremental AUC for blood glucose concentration (d, h). *n* = 6 mice per group. Analysis by ANOVA. ^^^Alb^−/−^ significantly different from WT (main effect of genotype, *p* < 0.05). For OGTT, post hoc testing indicated significant difference at every time point, while for ITT individual time points were not tested statistically because there was no significant interaction with the time factor in ANOVA. *Females significantly lower than males (main effect of sex, *p* < 0.05)

To test markers of insulin sensitivity, OGTT and ITT assessments were performed on live mice. In the analysis of blood glucose concentration for the OGTT (Figure 3a–d), three‐way ANOVA indicated a main effect of sex (*p* < 0.05), main effect of genotype (*p* < 0.05), main effect of time (*p* < 0.05), genotype‐by‐time interaction (*p* < 0.05), and no significant sex‐by‐genotype or sex‐by‐genotype‐by‐time interaction. Thus, the relative effect of albumin deficiency did not depend upon sex. Post hoc testing was performed to explore the genotype‐by‐time interaction, and this test indicated a significantly lower blood glucose concentration in Alb^−/−^ than WT at all time points (*p* < 0.05). AUC and incremental AUC (I‐AUC) were then analyzed by two‐way ANOVA. For AUC, ANOVA of the OGTT data (Figure 3c) indicated a significant main effect of sex (*p* < 0.05), main effect of genotype (*p* < 0.05), and no significant interaction. For I‐AUC (Figure 3d), only a main effect of genotype was observed for the OGTT response (*p* < 0.05). AUC and I‐AUC for blood glucose in the OGTT were significantly lower in Alb^−/−^ than WT. In the analysis of the ITT data, three‐way ANOVA on blood glucose over time (Figure 3e,f) indicated a main effect of sex (*p* < 0.05), main effect of genotype (*p* < 0.05), main effect of time (*p* < 0.05), and no significant interactions. The main effect of genotype corresponded to significantly lower blood glucose during the ITT in Alb^−/−^ as compared with WT. As with OGTT, ANOVA results for ITT indicated that the relative effect of albumin deficiency did not depend upon sex. AUC (Figure 3g) and I‐AUC (Figure 3h) for ITT were then analyzed by two‐way ANOVA. For AUC, ANOVA indicated a significant main effect of sex (*p* < 0.05), main effect of genotype (*p* < 0.05), and no significant interaction. AUC for blood glucose in the ITT was significantly lower in Alb^−/−^ than WT, and this genotype difference did not depend upon sex. In the ANOVA for I‐AUC results from the ITT, there were no significant main effects or interaction; thus, the effect of albumin deficiency upon blood glucose following insulin injection was related to the lower fasting blood glucose in Alb^−/−^. The differences between groups in blood glucose during OGTT and ITT were not a result of fasting insulin levels, as we did not observe any significant main effects or interactions in the analysis of fasting plasma insulin (WT male, 1.04 ± 0.08, Alb^−/−^ male, 1.16 ± 0.32, WT female, 0.86 ± 0.24, Alb^−/−^ female, 0.70 ± 0.10 µg/L).

**FIGURE 4:**
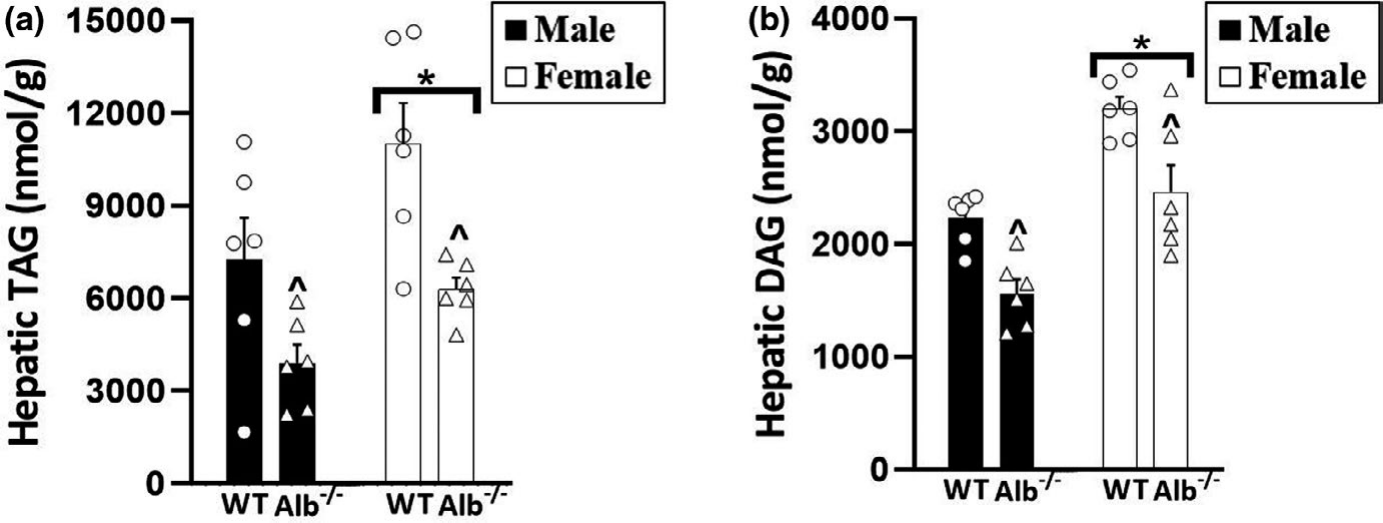
(a) Triacylglycerol (TAG) and (b) diacylglycerol (DAG) concentration in liver. *n* = 6 mice per group. Analysis by ANOVA. ^Alb^−/−^ significantly different from WT (main effect of genotype), *p* < 0.05. *Females significantly higher than males (main effect of sex, *p* < 0.05)

To determine if reduced plasma FFA concentration may lead to lower ectopic lipid deposition, specific lipids were measured in the liver. For hepatic TAG content (Figure 4a), a significant main effect of genotype (*p* < 0.05) and main effect of sex (*p* < 0.05) was observed with no significant interaction. The main effect of genotype corresponded to lower hepatic TAG in Alb^−/−^ than WT, while the main effect of sex corresponded to overall higher levels in females than males. For hepatic DAG content (Figure 4b), a significant main effect of genotype (*p* < 0.05) and main effect of sex (*p* < 0.05) was observed with no significant interaction. The main effect of genotype corresponded to lower hepatic DAG in Alb^−/−^ than WT, while the main effect of sex corresponded to overall higher levels in females than males. For hepatic ceramide concentration, no significant main effects or interaction were observed in the analysis (WT male, 89 ± 9; Alb^−/−^ male, 103 ± 5, WT female, 68 ± 10; Alb^−/−^ female, 86 ± 5 nmol/g). For hepatic CE, a significant main effect of sex (*p* < 0.05) indicated significantly higher CE in females than males; however, no significant main effect of genotype nor any significant interactions were observed for hepatic CE (WT male, 495 ± 49; Alb^−/−^ male, 651 ± 49, WT female, 841 ± 197; Alb^−/−^ female, 1047 ± 83 nmol/g). While the focus in lipid analysis was on the liver, to test if ectopic deposition in muscle exhibited similar responses, TAG was measured in skeletal muscle; for TAG in skeletal muscle, there was no significant main effects of sex or genotype, and no significant interaction (WT male, 3114 ± 762; Alb^−/−^ male, 3397 ± 988, WT female, 5655 ± 1155; Alb^−/−^ female, 4703 ± 902 nmol/g).

**FIGURE 5:**
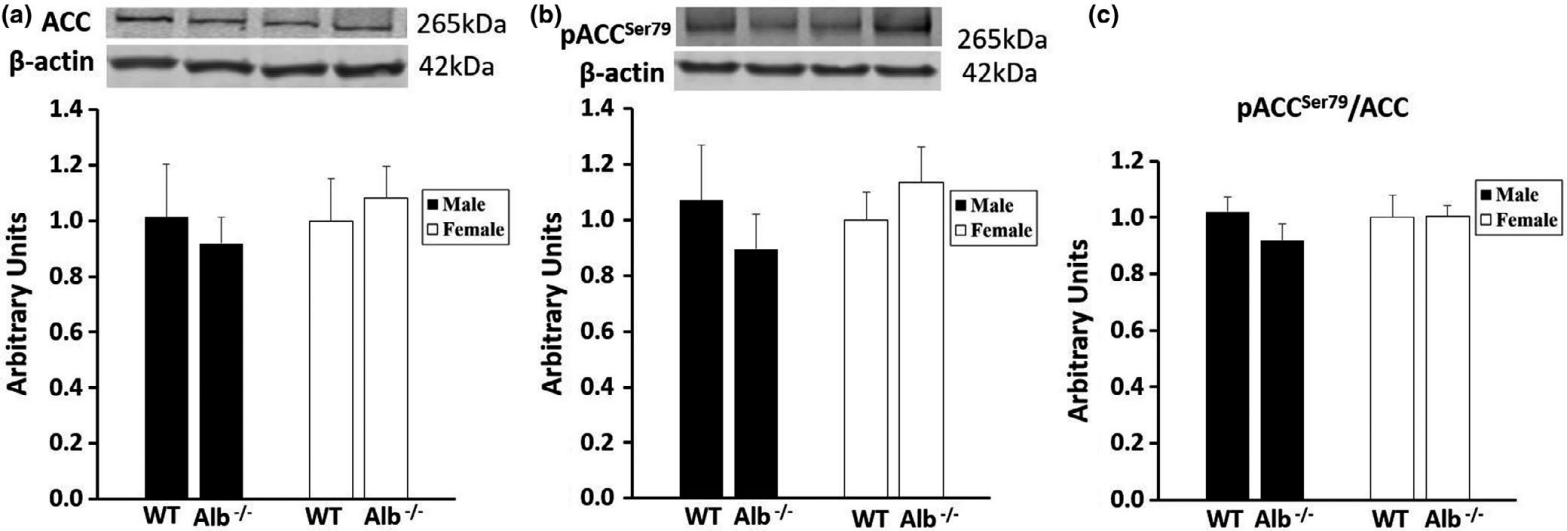
Acetyl CoA carboxylase (ACC) in liver. (a) Western blot for total ACC, (b) phosphorylated ACC, and (c) the ratio of phosphorylated to total ACC. *n* = 6 mice per group. Band intensity for each protein of interest was normalized to band intensity for β‐actin, and the arbitrary units scale was adjusted such that average WT female would be a value of 1. Analysis by ANOVA. No significant differences between groups

**FIGURE 6:**
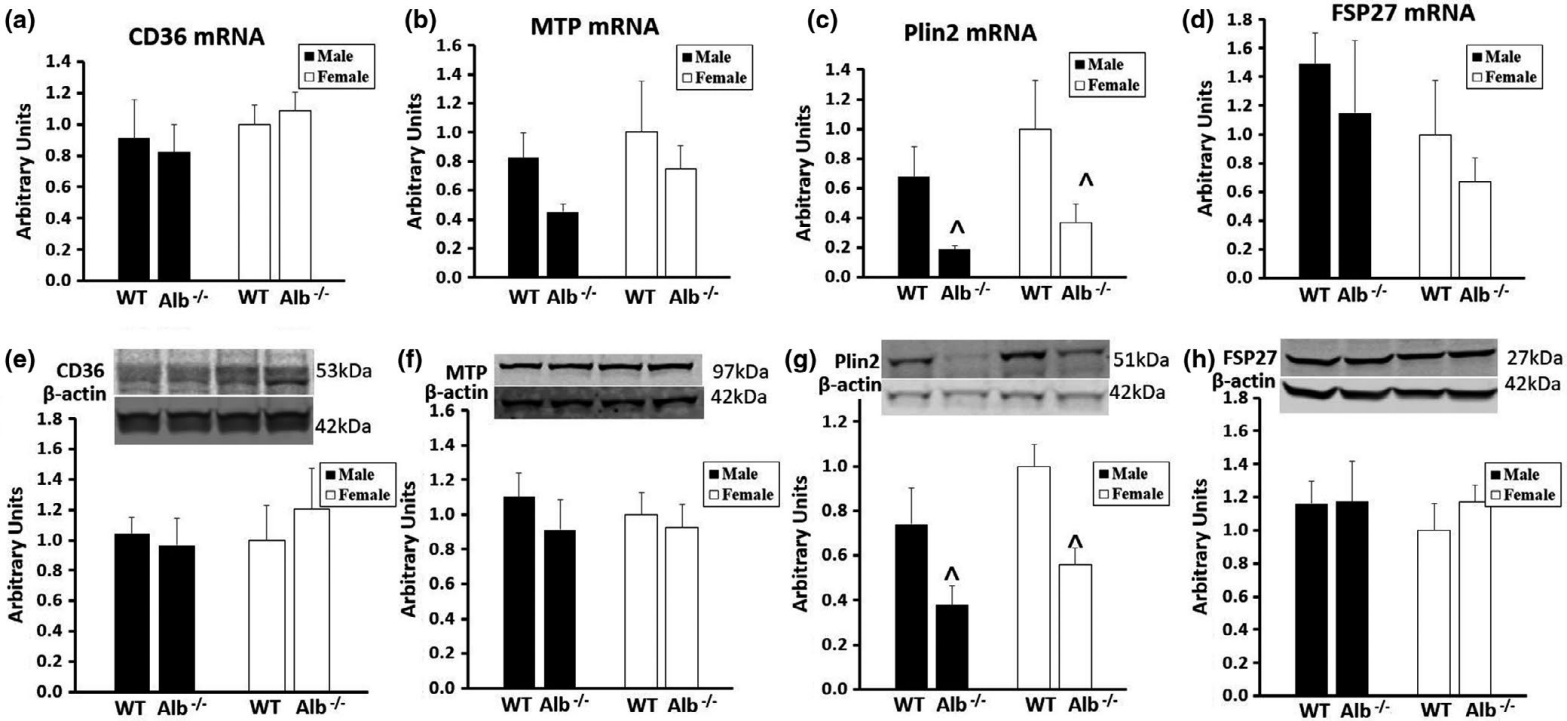
Gene expression (a–d) and protein expression (e–h) in liver. *n* = 6 mice per group. Gene expression normalized to 18S. Band intensity for each protein of interest was normalized to band intensity for β‐actin, and the arbitrary units scale was adjusted such that average WT female would be a value of 1. Analysis by ANOVA. ^Alb^−/−^ significantly different from WT (main effect of genotype, *p* < 0.05)

To understand metabolic responses in the liver, we performed measurements of gene and protein expression on this tissue. Total ACC expression (Figure 5a), phosphorylated ACC (Figure 5b), and the ratio between these two values (Figure 5c) in liver were not significantly different between groups. Western blot was also performed for proteins that are shown in Figure 6. Out of the proteins measured by western blot, only Plin2 (Figure 6g) was significantly different between groups. Plin2 gene expression (Figure 6c) changes corresponded with changes in protein expression, indicating potential control at the transcriptional level. For Plin2 gene expression (Figure 6c) and protein expression (Figure 6g), significant main effects of genotype (*p* < 0.05) were observed, with no significant main effects of sex or interactions. The main effects of genotype corresponded to lower Plin2 gene and protein expression in the liver of Alb^−/−^ compared to WT. There were no differences between groups for other gene and protein expression levels that are reported in Figure 6. Additionally, we observed no significant differences between groups in analyses of western blot data for FASN or ACLY (data not shown).

**FIGURE 7:**
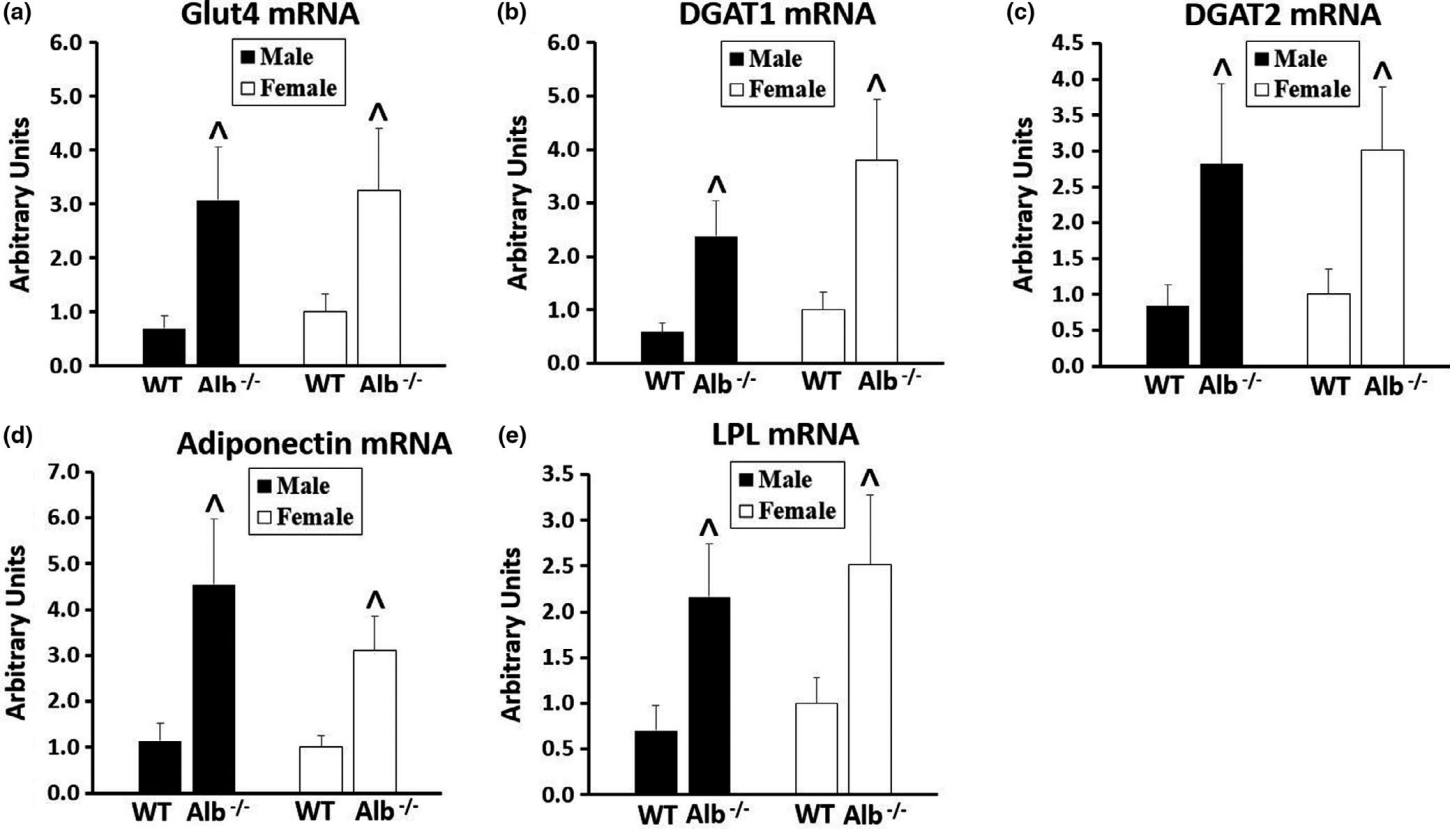
Gene expression (a‐e) in adipose tissue. *n* = 6 mice per group. Gene expression normalized to 18S. Analysis by ANOVA. ^Alb^−/−^ significantly different from WT (main effect of genotype, *p* < 0.05)

**FIGURE 8:**
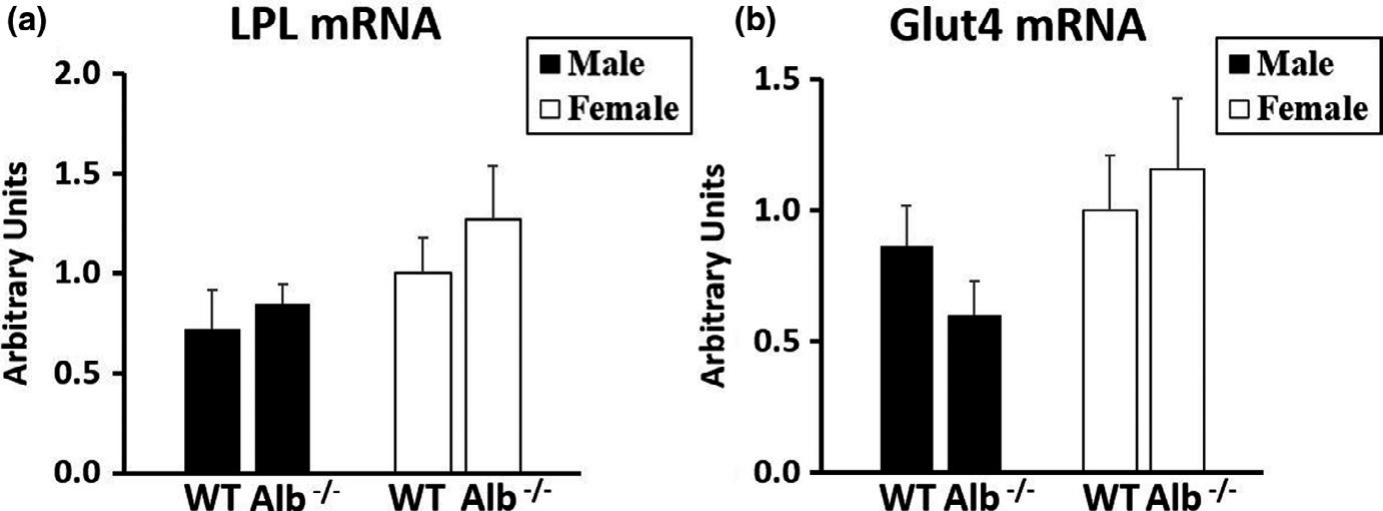
Gene expression (a, b) in skeletal muscle. *n* = 6 mice per group. Gene expression normalized to 18S. Analysis by ANOVA. No significant differences between groups

Because albumin deficiency could alter physiology of adipose tissue through reduction of FFA export efficiency, we tested gene expression levels in adipose tissue. Insufficient sample remained for western blotting on adipose tissue. As shown in Figure 7, for each gene expression level that was measured (Glut4, DGAT‐1, and ‐2, adiponectin, LPL), a significant main effect of genotype (*p* < 0.05) was observed with no significant main effects of sex or interaction. The main effects of genotype corresponded to higher expression of each gene in adipose tissue of Alb^−/−^ compared to WT. Gene expression for LPL (Figure 8a) and Glut4 (Figure 8b) were also measured in skeletal muscle, but no significant main effects of sex, genotype, or interaction were observed for these gene expression levels in muscle.

**FIGURE 9:**
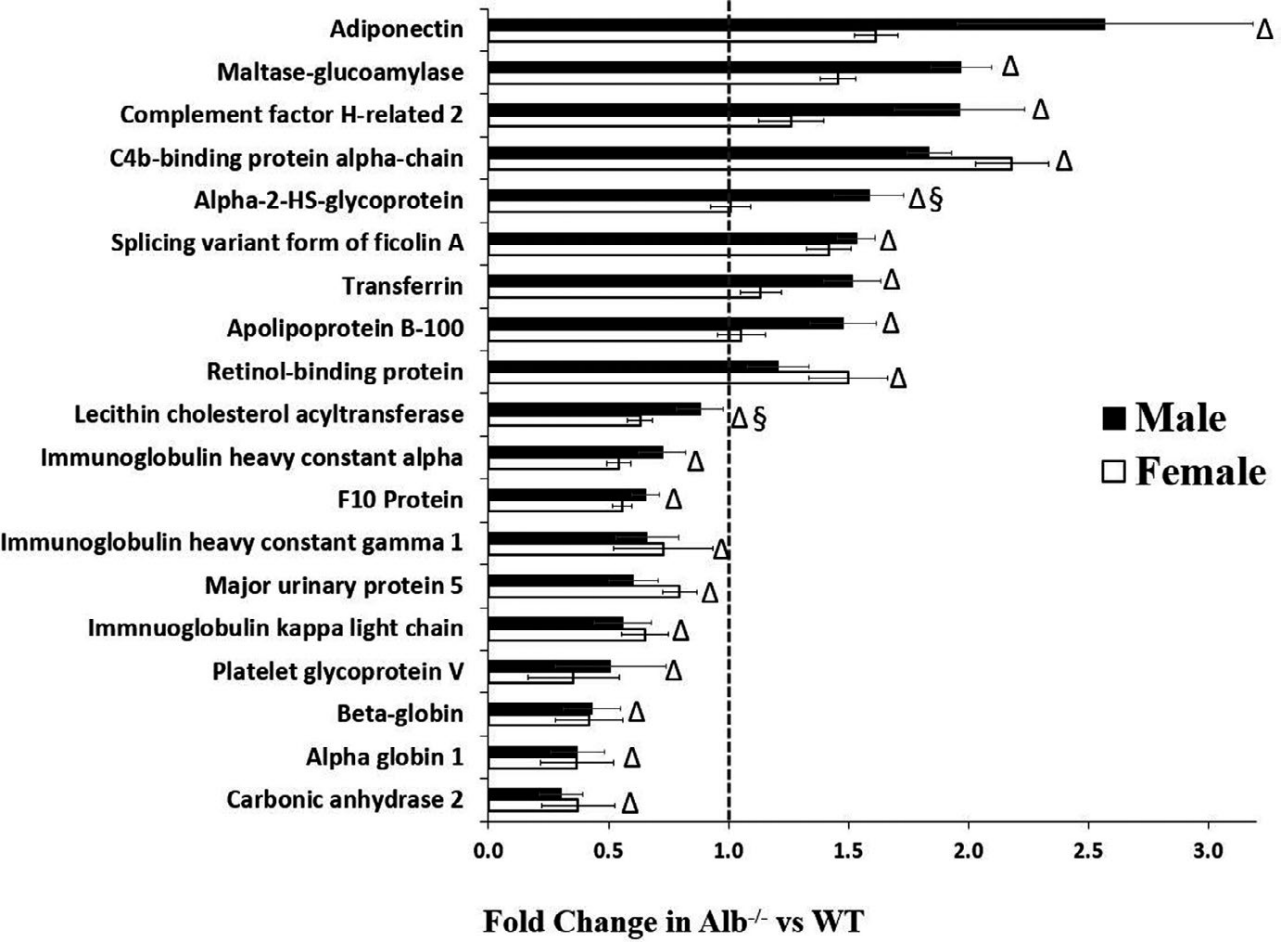
Plasma proteome. Proteins listed in the figure exhibited at least a 1.5‐fold difference between WT and Alb^−/−^. *n* = 6 mice per group. By two‐way ANOVA (genotype × sex), each of the proteins listed in the figure achieved a significant main effect of genotype (*p* < 0.05); LCAT and alpha‐2‐HS‐glycoprotein also exhibited a genotype‐by‐sex interaction, and post hoc testing indicated that each of these two proteins was only significantly altered in one sex. Δ, significant main effect of genotype. §, significant sex‐by‐genotype interaction. Fold‐change > 1 indicates albumin knockout higher than WT. Fold‐change of 0.67 is equivalent to a 1.5‐fold change for protein that exhibited reduced expression in Alb^−/−^

Total plasma protein concentration was analyzed by two‐way ANOVA, indicating a significant main effect of genotype (*p* < 0.05) but no significant main effect of sex or interaction; as potentially expected, total plasma protein was significantly lower in Alb^−/−^ than WT (WT male, 65 ± 2, Alb^−/−^ male, 40 ± 4, WT female, 60 ± 3, Alb^−/−^ female, 37 ± 1 g/L). In addition to the expected albumin deficiency in Alb^−/−^, the data filtering approach of the plasma proteomics data led to discovery of 19 plasma proteins exhibiting significantly altered expression in Alb^−/−^ compared to WT (Figure 9). The most significantly elevated protein was adiponectin, and this finding is consistent with gene expression results in adipose tissue that are described above. Two proteins were initially identified as “Igh” in Uniprot; a Blast search indicated that one was likely to be immunoglobulin heavy constant alpha (Igha) while the other was likely to be immunoglobulin heavy constant gamma 1 (Ighg1), based upon >99% sequence homology. Thus, in Figure 9 these proteins are identified as Igha and Ighg1.

## 4 DISCUSSION

Reducing the concentration of plasma FFA could change metabolism in various tissues and could be expected to improve insulin sensitivity. Specifically, slowing export of FFA from adipose tissue could reduce storage of lipid in other tissues, and we had hypothesized that impeding FFA‐albumin binding would decrease plasma FFA concentration and thus improve glucose tolerance and the response to insulin. Overall, the results confirmed our hypothesis. As binding of FFA with serum albumin is the final step in their export from adipose tissue, we tested whether albumin gene knockout could reduce plasma FFA concentration. We further tested if any FFA reduction would lead to impacts upon metabolic health. We examined the impact of albumin deficiency upon plasma FFA concentration, insulin sensitivity, storage of lipid in liver and muscle, whole body fuel selection, gene expression in various tissues, as well as protein expression in the liver and in plasma. Our novel findings include discoveries of reduced plasma FFA concentration, as well as reduced hepatic TAG and DAG, in male and female albumin knockout mice. We observed that this reduction of FFA and hepatic lipids is associated with improvements in glucose tolerance and enhanced response to insulin administration. Changes in adipose tissue gene expression were consistent with enhanced TAG‐FFA substrate cycling in this tissue. Furthermore, while reducing plasma FFA led to improved insulin sensitivity and reduced hepatic lipids, remarkably the FFA supply to tissues in albumin knockout mice remained sufficient for sustained whole body fat oxidation and normal exercise capacity. The findings have implications for the control of metabolism as well as for identification of potential therapeutic targets for modulating lipid and glucose metabolism.

A fundamental goal of the study was to ascertain if reduced albumin concentration could lead to reduced plasma FFA. In the initial report on the albumin knockout mouse model (Roopenian et al., 2015), it was suggested that plasma FFA concentration may be reduced by albumin deficiency, although the nutritional state of the mice was unclear. In addition to confirming that plasma FFA would be lower in albumin knockout mice than control mice, it was also a primary goal of our present investigation to test if the FFA reduction could result in reduced hepatic TAG. When we discovered reduced hepatic TAG alongside reduced plasma FFA, it seemed reasonable to infer that the reduced plasma FFA was the cause of the reduced hepatic TAG, through reduced FFA uptake in the liver. However, it could also be possible that changes in lipolysis, fat oxidation, or VLDL secretion in the liver would have been a cause of the altered lipid content. Thus, we tested expression of a lipid droplet protein involved in lipolysis and lipid droplet stability (FSP27) as well as a protein involved in VLDL assembly (MTP); expression level of these proteins was not altered in albumin knockout mice. We also tested ACC phosphorylation, an indicator of ACC activity, as ACC exerts control over de novo lipogenesis and fat oxidation. One would expect lower ACC activity if its relative phosphorylation level was higher, because AMPK phosphorylates ACC as a means of decreasing its activity (Tuazon et al., 2015, 2018). Nonetheless, ACC protein expression and phosphorylation were not different between groups. Although it is possible that other untested protein expression levels might be altered in this mouse model, at the present time we infer that the reduced plasma FFA concentration remained as a likely cause of the reduced hepatic TAG content. That is to say, reduced plasma FFA concentration potentially decreased FFA uptake through a reduced FFA concentration gradient. In addition to changes in hepatic TAG, hepatic DAG content was also lower in albumin knockout mice compared to WT. DAG is typically elevated when TAG is elevated (Camporez et al., 2015; Hall et al., 2014; Leavens et al., 2009; Puri et al., 2007; Wendel et al., 2010), and it is believed to lead to insulin resistance (Camporez et al., 2015; Perry et al., 2014; Samuel & Shulman, 2018). Though we measured total DAG, specifically a membrane bound DAG pool may lead to activation of protein kinase C, as a mechanism to promote insulin resistance (Camporez et al., 2015; Perry et al., 2014; Samuel & Shulman, 2018). Despite the likelihood of different DAG pools within hepatocytes, the statistically significant change in total liver DAG corresponded to our observation of improved insulin sensitivity in albumin knockout mice. Thus, ultimately FFA reduction, and its impacts on hepatic DAG, may have led to improved insulin sensitivity in albumin knockout mice.

Various populations would benefit from improved insulin sensitivity, such as obese people (Carr et al., 2016; Fabbrini et al., 2010; Kivimaki et al., 2017; Samuel & Shulman, 2018; Younossi et al., 2019), sleep apnea patients (Barcelo et al., 2011; Borel, 2019; Gu et al., 2017; Kamble et al., 2020), sleep‐deprived people (Borel, 2019; Broussard et al., 2015; Rao et al., 2015; Zhu et al., 2019), elderly (Milanesi & Weinreb, 2000; Takeda et al., 2011), spinal cord injury patients (Nash & Bilzon, 2018; Nash & Gater, 2020), and cigarette smokers (Haj Mouhamed et al., 2016; Hamabe et al., 2011; Rincon et al., 1999; Targher et al., 1997). Future work could focus on any of these populations which experience high incidence of pathologies and impaired insulin sensitivity. Here, we showed improvement in insulin sensitivity in healthy animals when albumin deficiency reprogramed lipid metabolism and ultimately impacted glucose metabolism. Reduced Glut4 expression in adipose tissue and skeletal muscle in insulin resistance has been reported (Kampmann et al., 2011; Leguisamo et al., 2012), and increase Glut4 expression in either tissue could be beneficial to health. We report elevated adipose tissue Glut4 gene expression in albumin knockout mice, and this change in adipose tissue biology may partially underlie the improvement in glucose tolerance and insulin response. In contrast, skeletal muscle Glut4 gene expression was not different between genotypes in the present study, indicating that the metabolic benefits in albumin knockout mice may primarily occur outside of skeletal muscle. This viewpoint is consistent with the observation that TAG was also not altered in skeletal muscle in albumin knockout mice in the present work. Overall, the observations indicate that the metabolic alterations in albumin knockout mice may be primarily centered around adipose tissue and liver rather than skeletal muscle. The superior insulin sensitivity in albumin knockout mice may be through changes in hepatic lipid metabolism, such as reduced lipotoxicity.

By reducing plasma FFA carrying capacity in the albumin knockout mouse, the proximal step in lipid mobilization (lipolysis) is expected to remain intact, while FFA transport from cells to the bloodstream is slowed. This slowed export is expected because dissolution of FFA into the bloodstream is the final step of export from adipose tissue, but this dissolution relies upon albumin. While one might expect that reduced plasma FFA supply to tissues could lead to difficulty in maintaining the whole body metabolic rate and fat oxidation, remarkably we observed that the albumin knockout mice did not exhibit any depression in metabolic rate or fat oxidation. Thus, though the reduced plasma FFA concentration was sufficient to decrease hepatic lipids, it appears to have remained high enough to fuel resting substrate oxidation.

Compensation for the slowed release of FFA from adipocytes could be through enhanced intracellular FFA re‐esterification within the adipocytes. Intracellular FFA re‐esterification via futile cycling in adipose tissue is an ongoing metabolic process in healthy individuals (Henderson et al., 2007; Miyoshi et al., 1988; Wolfe & Peters, 1987). This natural metabolic pathway would likely be enhanced in the albumin knockout mouse such that lipolysis could proceed at a reasonable rate with the majority of released FFA recycled back into the lipid droplet. Through ex vivo studies of adipose tissue (Chitraju et al., 2017; Leibel et al., 1985; Nye et al., 2008; Vaughan, 1962; Wood et al., 1960), as well as through work in humans with isotope tracers in vivo (Henderson et al., 2007; Miyoshi et al., 1988; Wolfe & Peters, 1987), it has been established that the rate of intracellular lipolysis typically exceeds the rate of FFA release because of immediate re‐esterification of FFA within adipocytes. Furthermore, the relative rate of this TAG‐FFA cycle exhibits substantial plasticity, with potential for either low or very high relative rates of re‐esterification within the adipocyte, as indicated by in vivo studies (Henderson et al., 2007; Miyoshi et al., 1988; Wolfe & Peters, 1987) and in vitro studies of isolated adipose tissue (Chitraju et al., 2017; Leibel et al., 1985; Nye et al., 2008; Vaughan, 1962; Wood et al., 1960). Thus, we expect that adipose tissue can accommodate the albumin deficiency in albumin knockout mice through a compensatory increase in the rate of TAG‐FFA cycle activity, and this view is supported by the present results related to adipose tissue gene expression. Diacylglycerol acyltransferase (DGAT‐1 and ‐2), which are involved in TAG synthesis and TAG‐FFA cycling, exhibited significantly higher gene expression in albumin knockout than WT. Glut4 expression was also significantly elevated in albumin knockout mouse adipose tissue compared with WT, and this adaptation may support glycerol‐3‐phosphate generation for the high TAG‐FFA cycle activity. The elevated Glut4 expression in adipose tissue also agrees with the improved insulin sensitivity that was observed in this model in comparison to WT. Other changes observed in adipose tissue of albumin knockout mice were the elevated LPL and adiponectin gene expression. The elevation of LPL gene expression (if it ultimately translates to increased LPL protein expression and activity) could allow potentially for sustained lipid uptake into the tissue when plasma FFA concentration is reduced. The elevated adiponectin gene expression corresponds to findings from the plasma proteome analysis, as discussed below.

The global proteome analysis of plasma indicated some changes in the plasma protein distribution, and these alterations included higher plasma adiponectin abundance in albumin knockout mice. Adiponectin promotes insulin sensitivity (Berg et al., 2001; Yamauchi et al., 2001), and this is consistent with superior insulin sensitivity in albumin knockout mice than WT. It is also noteworthy that retinol‐binding protein (RBP4) and alpha‐2‐HS‐glycoprotein (fetuin‐A) were each elevated in albumin knockout mice, as each of these proteins are known to bind FFA (Cayatte et al., 1990; Pal et al., 2012; Perduca et al., 1863, 2018); increased expression may be a compensatory mechanism to counteract reduced plasma FFA: however, these changes are not sufficient to maintain plasma FFA at levels of WT mice. Finally, we note C4b‐binding protein was elevated in albumin knockout mice compared with WT, and this protein has been shown to be protective against type 2 diabetes (Ermert & Blom, 2016; Kulak et al., 2017; Sjolander et al., 2016). Both the changes in adipose tissue biology and the plasma proteome could be involved in determining the phenotype of albumin knockout mice. Yet also, it is expected that the reduced hepatic lipids (TAG and especially DAG) could be an underlying factor in the improved insulin sensitivity.

The present results indicate that albumin could theoretically be a therapeutic target to reduce plasma FFA concentration and improve insulin sensitivity. In the future if therapeutics for impeding FFA binding to albumin were developed, it would be reasonable to hypothesize that this intervention could lead to reduced plasma FFA concentration, reduced hepatic lipids, and improved insulin sensitivity. That is to say, one could consider the albumin knockout mouse model to be a laboratory tool for understanding whether reducing the binding of FFA to serum albumin could lead to favorable outcomes. Indeed the insulin sensitivity improvements that are expected from reducing plasma FFA concentration (Henderson, 2021) are seen in the albumin knockout mouse. Nonetheless, the phenotype is not entirely limited to FFA trafficking, and we also note that this mouse model exhibits reduced total plasma protein concentration; this observation is not surprising, as albumin is the most abundant protein in plasma in healthy individuals. Congenital analbuminemia (CAA), resulting from an individual being homozygous for albumin gene mutation, is extremely rare in humans. Case reports on CAA patients indicate that their total plasma protein concentration is often considered to be below the standard reference range (Caridi et al., 2008; Koot et al., 2004), in agreement with the mouse model. Adults with CAA are not widely considered to have an altered life expectancy, and the patients can be nearly asymptomatic, with the CAA diagnosis sometimes being by chance through routine lab work (Caridi et al., 2012; Koot et al., 2004). Others have been discovered to have CAA because they exhibited mild or moderate edema, which is an apparent symptom of CAA in some but not all patients (Caridi et al., 2008; Dammacco et al., 1980). Unfortunately, plasma FFA and glucose results have not been reported in most case reports of CAA, and thus the sample size is far too limited to draw a conclusion about these parameters in humans with the condition.

It has been speculated that ApoB and its related lipoproteins are elevated in CAA patients, based upon comparison to reference ranges (Del Ben et al., 2013; Weigand et al., 1983); however, these patients were not compared to appropriately matched control subjects. As is common practice in case reports, the results for patients were compared to clinical reference ranges. Indeed, many people these days, expressing normal albumin levels, do exhibit some elevation of ApoB (and a related elevation of plasma cholesterol and TAG), as a result of an unhealthy diet and elevated bodyweight. Nonetheless, the present results from albumin knockout mice confirm that albumin deficiency per se may lead to elevated plasma ApoB, plasma TAG, and TC. It is also worth noting that HDL‐C is proportionally increased with TC in the mouse model, which may limit any detriments of elevated ApoB‐related lipoproteins. It is also noteworthy that low plasma FFA concentration and high insulin sensitivity are considered to be factors that would exert benefits upon cardiovascular health. Indeed, high plasma FFA is believed to worsen all‐cause mortality rate and CVD‐related mortality in humans (Henderson, 2021). High plasma FFA abundance promotes heart disease and vascular dysfunction, through direct effects of the plasma FFA abundance upon the heart and blood vessels (Henderson, 2021). Thus, long‐term reduction of plasma FFA concentration would be expected to reduce mortality risk. Furthermore, insulin resistance is associated with a significant increase in CVD incidence and mortality (Moshkovits et al., 2021), and therefore the high insulin sensitivity (e.g., as seen in the albumin knockout mouse) may be protective from CVD. Thus, while the phenotype of albumin deficiency is complex, we infer that the changes in plasma FFA trafficking and insulin sensitivity seen with albumin deficiency in the albumin knockout mouse may represent a phenotype that would be generally beneficial to cardiometabolic health. In therapeutic development in the future, it would be important to harness the improvements in FFA metabolism that are observed in the mouse model, while leaving the total plasma protein concentration in the normal range to avoid any undesirable effects exerted by decreased plasma protein concentration.

## 5 SUMMARY AND CONCLUSION

Plasma FFA elevation is a risk factor for various diseases, and generally reducing plasma FFA in both healthy and unhealthy individuals is expected to improve metabolic well‐being (Henderson, 2021). We have demonstrated an important role of serum albumin in determining plasma FFA concentration. It is generally expected that decreasing plasma FFA can improve insulin sensitivity, and we have shown that albumin knockout mice indeed exhibit reduced plasma FFA concentration alongside improved glucose tolerance and insulin sensitivity. This finding suggests that the albumin knockout mouse could be a useful tool for studies of the relationship between plasma FFA and metabolic health. Therefore, to set the stage for future research on this model, we broadly characterized the in vivo phenotype, gene and protein expression, lipids, and the plasma proteome. This critical work was performed on mice that consumed a standard diet, rather than a diet that was specifically high in a deleterious nutrient class such as high fat or high sugar. The present findings are expected to be supportive of future work conducted under conditions of specific pathologies. For example, chronic obesity, cigarette smoking, sleep apnea, sleep restriction, and surely other pathological factors lead to elevated plasma FFA and a resulting impairment of lipid and glucose metabolism (Henderson, 2021). Future work could build upon our present findings by applying this mouse model to any of these pathological health factors. For example, we note that obese mice could be studied in order to test if reduced plasma FFA through targeting albumin could lead to a reduced propensity toward diabetes and fatty liver disease. Finally, we note that FFA is not the only ligand for circulating albumin, although it is arguably the primary ligand. The phenotype of albumin knockout mice is clearly centered on FFA metabolism, but changes in abundance or bioavailability of other compounds, as well as remodeling of the proteome, could also impact metabolism under conditions of albumin deficiency. Nonetheless, we observed insulin sensitivity enhancement that corresponded to the reduced plasma FFA concentration in this mouse model, and studying this model could thus lead to insights that inform development of therapeutics. Ultimately, for translating this line of research to clinical impact, methods of targeting albumin, and its FFA binding would be needed that can exert their actions without actually changing hepatic albumin expression; this could involve developing inhibitors of FFA binding to albumin, such that albumin‐FFA binding could be targeted for metabolic health improvement without reducing the circulating albumin concentration. In summary, the present findings indicate that plasma FFA concentration is reduced in albumin knockout mouse, and this FFA suppression is associated with favorable changes in insulin sensitivity and reduced hepatic lipids.

## CONFLICT OF INTEREST

The authors declare no conflict of interest.

## AUTHOR CONTRIBUTIONS

Gregory C. Henderson conception and design of research; Afsoun Abdollahi, Brianna N. Dowden, Kimberly K. Buhman, Alyssa S. Zembroski, and Gregory C. Henderson performed experiments; Afsoun Abdollahi, Brianna N. Dowden, and Gregory C. Henderson analyzed data; Afsoun Abdollahi, Brianna N. Dowden, and Gregory C. Henderson prepared figures; Gregory C. Henderson and Afsoun Abdollahi wrote the manuscript; Afsoun Abdollahi, Brianna N. Dowden, Kimberly K. Buhman, Alyssa S. Zembroski, and Gregory C. Henderson edited and revised the manuscript; Afsoun Abdollahi, Brianna N. Dowden, Kimberly K. Buhman, Alyssa S. Zembroski, and Gregory C. Henderson approved the final version of the manuscript.
